# Epidemiological Analysis of Trauma Patients following the Lushan Earthquake

**DOI:** 10.1371/journal.pone.0097416

**Published:** 2014-05-20

**Authors:** Li Zhang, Minggang Zhao, Wenhao Fu, Xinqiang Gao, Ji Shen, Zuyun Zhang, Ming Xian, Yunzhi Jiao, Jian Jiang, Jinqian Wang, Guomin Gao, Bin Tang, Liang Chen, Weimin Li, Changhua Zhou, Shaoping Deng, Jianwen Gu, Dong Zhang, Ying Zheng, Xiangmei Chen

**Affiliations:** 1 State Key Laboratory of Kidney Disease, Department of Nephrology, Chinese PLA General Hospital and Military Medical College, Beijing, China; 2 National Health and Family Planning Commission of the People’s Republic of China, Beijing, China; 3 Health and Family Planning Commission of Sichuan Province, Chengdu, China; 4 Department of Health, United Logistic Ministry, Chengdu Military Region, Chengdu, China; 5 West China Hospital, Sichuan University, Chengdu, China; 6 Public Health Bureau of Yaan City, Yaan, China; 7 Sichuan Provincial People’s Hospital, Chengdu, China; 8 General Hospital of Chengdu Military Command, Chengdu, China; The Ohio State University, United States of America

## Abstract

**Background:**

A 7.0-magnitude earthquake hit Lushan County in China’s Sichuan province on April 20, 2013, resulting in 196 deaths and 11,470 injured. This study was designed to analyze the characteristics of the injuries and the treatment of the seismic victims.

**Methods:**

After the earthquake, an epidemiological survey of injured patients was conducted by the Health Department of Sichuan Province. Epidemiological survey tools included paper-and-pencil questionnaires and a data management system based on the Access Database. Questionnaires were completed based on the medical records of inpatients with earthquake-related injuries. Outpatients or non-seismic injured inpatients were excluded. A total of 2010 patients from 140 hospitals were included.

**Results:**

The most common type of injuries involved bone fractures (58.3%). Children younger than 10 years of age suffered fewer fractures and chest injuries, but more skin and soft -tissue injuries. Patients older than 80 years were more likely to suffer hip and thigh fractures, pelvis fractures, and chest injuries, whereas adult patients suffered more ankle and foot fractures. A total of 207 cases of calcaneal fracture were due to high falling injuries related to extreme panic. The most common type of infection in hospitalized patients was pulmonary infections. A total of 70.5% patients had limb dysfunction, and 60.1% of this group received rehabilitation. Most patients received rehabilitation within 1 week, and the median duration of rehabilitation was 3 weeks. The cause of death of all seven hospitalized patients who died was severe traumatic brain injuries; five of this group died within 24 h after the earthquake.

**Conclusions:**

Injuries varied as a function of the age of the victim. As more injuries were indirectly caused by the Lushan earthquake, disaster education is urgently needed to avoid secondary injuries.

## Introduction

China is an earthquake-prone country. Three earthquakes with magnitudes above 7.0 have occurred in China in the past 5 years: the Wenchuan, Yushu, and Lushan earthquakes [1]–[3]. Earthquakes disasters often lead to mass casualties, severe bodily injuries, and psychological and mental disorders that have a substantial impact on the quality of life of survivors. At 08∶02 Beijing time on April 20, 2013, a 7.0-magnitude earthquake hit Lushan County in Ya’an city in south China’s Sichuan province. The epicenter, with a depth of 13 km, which is ∼110 km from Chengdu. As of 14∶30 on April 24, a total of 196 people had been confirmed dead, 21 had been confirmed missing, and 11470 had been confirmed injured [4], [5].

Based on the experience gained from the 2008 Wenchuan earthquake, the Chinese government and various departments responded quickly to this earthquake. Eighteen minutes after the quake hit, the Chengdu Military Region organized the earthquake relief headquarters, and the China Earthquake Administration immediately launched a first-grade emergency response to the earthquake [5]. Five hours after the quake hit, the Health Department of Sichuan Province dispatched nearly 200 medical staff from 12 medical corps to the front line of the quake-relief work to engage in emergency rescues. The National Health and Family Planning Commission dispatched five medical rescue teams and one expert medical team to manage medical relief efforts in the quake-affected areas. The expert team was composed of experts in intensive care, orthopedics, general surgery, neurosurgery, thoracic surgery, pediatric surgery, infection, nephrology, and rehabilitation. The “four centralization” principles (i.e., centralize the victims, medical experts, equipment, and treatment) were followed during the rescue operations, and daily reports of the condition of those who were severely wounded were provided [6].

One week after the earthquake, the expert team developed a questionnaire and a data management system to epidemiologically survey inpatients with injuries related to the Lushan earthquake. This study was conducted to comprehensively analyze earthquake-related injuries, examine the status of rescue personnel and material support, and provide data to assist planning for future earthquake disaster relief.

## Materials and Methods

### Ethics Statement

The study protocol was approved by the Ethics Committee of the Chinese PLA General Hospital, and the need for informed consent was waived. Patient records/information were anonymized and de-identified prior to analysis.

### Epidemiological Investigation

After the Lushan earthquake, the Health Department of Sichuan Province ordered all its subordinate municipal Health Administrative Departments to conduct an epidemiological investigation of all injured hospitalized patients. The epidemiological survey tools included paper-and-pencil questionnaires and a data management system based on the Access Database. All questionnaires and the Access database were designed by the expert team, and all investigators were trained. The primary data for the investigation included the hospital records of the injured patients, laboratory test results, and related rehabilitation records. The survey included all patients hospitalized for injuries caused by the Lushan earthquake; all outpatients and inpatients injured by other causes were excluded. The questionnaire consisted of seven parts. Part 1 contained general questions. Part 2 presented questions about overall treatment. Part 3 related to injuries and treatment. Part 4 covered rehabilitation. Part 5 included questions about infections during hospitalization and antimicrobial drugs used. Part 6 addressed complications experienced by patients during hospitalization. Part 7 examined laboratory results. The investigation was conducted from May 9, 2013 to July 9, 2013. All data entry was completed by August 14, 2013. All data are stored at the Information Center of the Health Department of Sichuan Province.

#### Definitions

*Critically ill:* Victims in a hemodynamically unstable condition with life-threatening organ dysfunction or/and severe infection who met ICU admission criteria.

*Seriously ill:* Victims with a potential risk of organ dysfunction or critical illness who required close observation [7].

### Data Collection

All data collected via the questionnaires were entered into the Access database and then converted into Excel files for processing. A total of 2010 patients were drawn from 140 hospitals. 155 patients were transferred, 141 of whom were hospitalized twice, and 14 of whom were hospitalized three times. To avoid repetition, duplicate records were combined. All data were checked by the two authors independently.

### Statistical Analysis

Descriptive statistics for all numerical variables, including the means, standard deviations, and percentages of all categorical variables, were calculated. The Kolmogorov-Smirnov test was used to determine the normality of the distribution of continuous variables. Continuous variables with normal distributions were expressed as means ± standard deviations, otherwise, medians and interquartile ranges (IQRs) were used. Categorical variables were expressed as absolute values and percentages. Patients were divided into nine groups according to age: ≤10, 11–20, 21–30, 31–40, 41–50, 51–60, 61–70, 71–80, and 81–94. Categorical variables were compared using *χ* ^2^ tests. All *P* values were two-tailed, and values <0.05 were considered to indicate statistical significance. The SPSS software 13.0 (Chicago, IL) was used for statistical analysis.

## Results

### Demographic Characteristics

**Figure 1:**
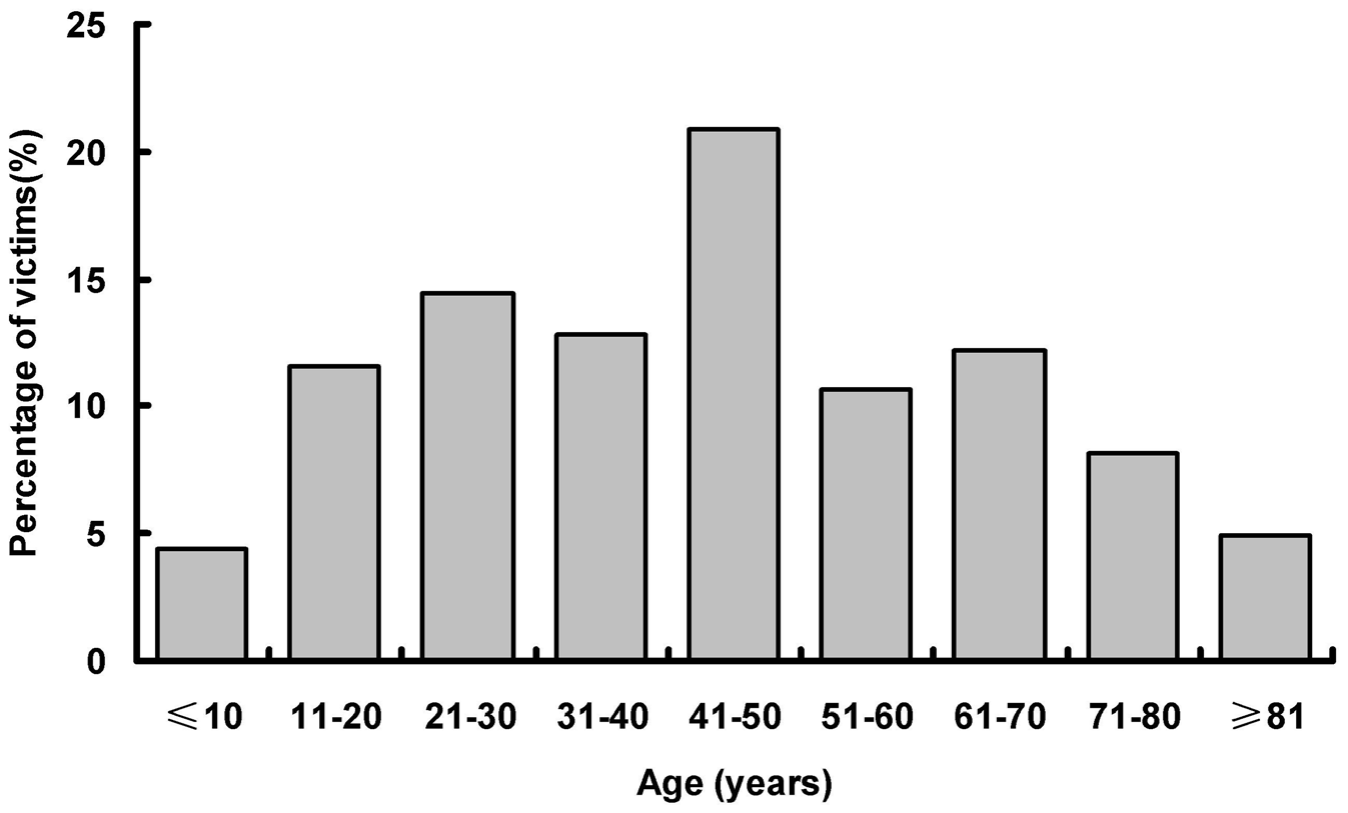
Age distribution of victims.

The mean age of the 2010 earthquake victims was 44.2±21.3 years, and 1129 (56.2%) were male. Children younger than 15 years accounted for 8.2% and those older than 60 years accounted for 25.2% of the victims (Figure. 1). The mean temperature on admission was 36.8±0.5°C, the mean heart rate was 82±13 beats per minute, the mean breath rate was 20±2 breaths per minute, and the mean systolic and diastolic blood pressure were 125.6±18.2 and 76.6±11.8 mmHg, respectively.

### Geographic Distribution of Injured Individuals

Lushan County in Ya’an City contained the largest number of injured (780/2010, 38.8%) and severely injured (105/333, 31.5%) victims; this was followed by Mingshan District, Yucheng District, Baoxing County, and Tianquan (Table 1).

**Table 1:** Geographical distribution of 2010 patients hospitalized following the Lushan Earthquake.

Injury locations	Injuries (%)	Critically ill and Seriously ill (%)
Ya’an Lushan	780 (38.8)	105 (31.5)
Ya’an Mingshan	201 (10.0)	21 (6.3)
Ya’an Yucheng	186 (9.3)	26 (7.8)
Ya’an Baoxing	135 (6.7)	42 (12.6)
Ya’an Tianquan	106 (5.3)	30 (9.0)
Ya’an Hanyuan	18 (0.9)	6 (1.8)
Ya’an Yingjing	13 (0.7)	2 (0.6)
Ya’an Shimian	9 (0.5)	1 (0.3)
Chengdu Qionglai	86 (4.3)	9 (2.7)
Chengdu Downtown	51 (2.5)	3 (0.9)
Chengdu Shuangliu	38 (1.9)	2 (0.6)
Chengdu Dayi	34 (1.7)	8 (2.4)
Chengdu Pujiang	32 (1.6)	6 (1.8)
Chengdu Others	19 (1.0)	11 (3.3)
Meishan	82 (4.1)	8 (2.4)
Deyang	63 (3.1)	10 (3.0)
Ziyang	32 (1.6)	3 (0.9)
Leshan	26 (1.3)	7 (2.1)
Zigong	10 (0.5)	3 (0.9)
Ganzi	7 (0.4)	3 (0.9)
Aba	4 (0.2)	2 (0.6)
Neijing	4 (0.2)	2 (0.6)
Liangshan	2 (0.1)	0 (0.0)
Mianyang	1 (0.1)	0 (0.0)
Guang’an	1 (0.1)	0 (0.0)
Unknown	70 (3.5)	23 (6.9)
Total	2010 (100.0)	333 (100.0)

### Analysis of Injuries

Of the 2010 patients admitted to hospitals, 1172 (58.3%) injuries involved fractures or crush injuries. The causes of injuries were jumping (371, 31.7%), smashing (239, 20.4%), falling (181, 15.4%), and crushing/cutting/unknown (381, 32.5%). Age-stratified profile of injuries and deaths are provided in Table 2. Children younger than 10 years suffered fewer fractures and chest injuries, but more skin and soft -tissue injuries. Patients older than 80 years were considerably more likely to suffer hip and thigh fractures, pelvis fractures, and chest injuries, whereas patients 11–50 years suffered more ankle, and foot fractures. There were no significant differences among all age groups in upper limb fractures, skull bone fractures, crush injuries, maxillofacial injuries, brain trauma, or abdominal injuries (Table 2). A total of 207 cases of calcaneal fractures due to high falling injuries were recorded. Thirty-five patients had injuries involving crushed limbs, 29 of whom had lower-extremity and six of whom had upper-extremity injuries. Traumatic shock occurred in 21 patients.

**Table 2:** Profile of injuries associated with the Lushan Earthquake by age group.

	Age Groups (years)	≤10	11∼20	21∼30	31∼40	41∼50	51∼60	61∼70	71∼80	≥81	P value
	Numbers of Patients	N (%)	N (%)	N (%)	N (%)	N (%)	N (%)	N (%)	N (%)	N (%)	
Fractures and crush injuries	1172	19(21.8)	130(56.3)	178(61.4)	163(63.4)	248(57.5)	131(61.5)	139(57.7)	103(63.2)	61(62.9)	<0.001
Limb fractures	829	13(14.9)	104(45)	130(44.8)	117(45.5)	179(41.5)	81(38.0)	90(37.3)	69(42.3)	46(47.4)	<0.001
Upper limb	240	6(6.9)	27(11.7)	30(10.3)	29(11.3)	48(11.1)	36(16.9)	33(13.7)	23(14.1)	8(8.2)	0.209
Lower limb	656	7(8.0)	88(38.1)	112(38.6)	100(38.9)	144(33.4)	51(23.9)	61(25.3)	51(31.3)	42(43.3)	<0.001
Hip/Thigh	130	2(2.3)	9(3.9)	13(4.5)	10(3.9)	26(6.0)	8(3.8)	16(6.6)	24(14.7)	22(22.7)	<0.001
Knee/Lowe leg	227	2(2.3)	28(12.1)	26(9.0)	40(15.6)	57(13.2)	24(11.3)	24(10.0)	14(8.6)	12(12.4)	0.033
Ankle/Foot	394	4(4.6)	64(27.7)	89(30.7)	65(25.3)	91(21.1)	23(10.8)	29(12.0)	18(11.0)	11(11.3)	<0.001
Spine fractures	323	1(1.1)	33(14.3)	61(21.0)	49(19.1)	61(14.2)	41(19.2)	40(16.6)	22(13.5)	15(15.5)	0.001
Pelvis fractures	70	1(1.1)	11(4.8)	11(3.8)	5(1.9)	17(3.9)	6(2.8)	8(3.3)	3(1.8)	8(8.2)	0.117
Rib/Sternal fractures	133	1(1.1)	5(2.2)	9(3.1)	14(5.4)	33(7.7)	26(12.2)	18(7.5)	18(11.0)	9(9.3)	<0.001
Skull bone fractures	84	4(4.6)	6(2.6)	10(3.4)	15(5.8)	20(4.6)	9(4.2)	10(4.1)	8(4.9)	2(2.1)	0.740
Crush injuries	71	4(4.6)	5(2.2)	11(3.8)	10(3.9)	18(4.2)	10(4.7)	6(2.5)	5(3.1)	2(2.1)	0.802
Brain injuries	84	6(6.9)	6(2.6)	10(3.4)	12(4.7)	20(4.6)	10(4.7)	10(4.1)	8(4.9)	2(2.1)	0.729
Maxillofacial injuries	81	5(5.7)	8(3.5)	8(2.8)	9(3.5)	26(6.0)	8(3.8)	8(3.3)	7(4.3)	2(2.1)	0.426
Chest injuries	104	1(1.1)	6(2.6)	10(3.4)	11(4.3)	32(7.4)	14(6.6)	9(3.7)	9(5.5)	12(12.4)	0.002
Abdominal injuries	31	3(3.4)	7(3.0)	5(1.7)	2(0.8)	7(1.6)	6(2.8)	0(0)	1(0.6)	0(0)	0.059
Skin and soft tissue injuries	761	61(70.1)	88(38.1)	109(37.6)	90(35.0)	162(37.6)	70(32.9)	95(39.4)	52(31.9)	34(35.1)	<0.001
Death	7	0	0	1(0.3)	2(0.8)	2(0.5)	1(0.5)	0	1(0.6)	0	-

### Infections

Infection sites were recorded for 143 patients during hospitalization (Table 3). Lung infections were the most common type of infection suffered by hospitalized patients (60, 37.7%); this was followed by skin and soft tissue infections (26, 16.4%) and secondary open wound infections (25, 15.7%). The mean age of the 60 victims with pulmonary infections was 57.8 years (median 65.5, IQR 37.7, range 0–95 years); eight (13.3%) were <2 and 37 (61.7%) were >60 years of age. Pathogens were detected in 54 of 143 patients (37.8%) (Table 4).

**Table 3:** Analysis of infection sites in the 143 patients hospitalized following the Lushan Earthquake.

Infection sites	Number (%)	Infection time			
		Number (%)	Mean±SD	Median(IQR)	Min-Max
Pulmonary infections	60 (37.7)	34 (40.0)	7.7±6.3	5.5(9)	0–23
Skin and soft tissue infections	26 (16.4)	14 (16.5)	4.3±4.5	3.5(8)	0–13
Open wounds infections	25 (15.7)	14 (16.5)	7.9±6.1	6(10)	0–23
Urinary tract infections	12 (7.5)	9 (10.6)	11.9±7.0	13(14)	2–20
Surgical site infections	6 (3.8)	4 (4.7)	11.0±5.5	11(10)	5–17
Abdominal cavity infections	4 (2.5)	2 (2.4)	9.5±5.0	NA	6–13
Gastrointestinal infections	3 (1.9)	NA	NA	NA	NA
Intracranial infections	2 (1.3)	2 (2.4)	10.5±9.2	NA	4–17
Catheter-related urinary tract infections	2 (1.3)	2 (2.4)	15.5±0.7	NA	15–16
Blood Infections	2 (1.3)	2 (2.4)	29.5±21.9	NA	14–45
Others					
Bronchitis	7 (4.4)	NA	NA	NA	NA
Oral infections	3 (1.9)	1 (1.2)	17	NA	NA
Upper respiratory tract infections	2 (1.3)	NA	NA	NA	NA
Acute cholecystitis	1 (0.6)	1 (1.2)	4	NA	NA
Traumatic Pancreatitis	1 (0.6)	NA	NA	NA	NA
Conjunctivitis	1 (0.6)	NA	NA	NA	NA
Traumatic iridocyclitis	1 (0.6)	NA	NA	NA	NA
Otitis media	1 (0.6)	NA	NA	NA	NA
Total	143 (100.0)	85 (100.0)			

**Table 4:** Pathogens identified in the 54 hospitalized patients with infections.

Pathogens	Infection sites								Total
	Open wounds	Surgical site	Intracranial	Lung	Urinary tract	Catheter-related urinary tract	Skin and soft tissue	Blood	
Escherichia coli	2	1	0	0	2	1	0	1	7 (13.0)
Pseudomonas aeruginosa	2	0	0	0	0	0	1	0	3 (5.6)
Staphylococcus haemolyticus	1	0	1	0	0	0	0	0	2 (3.7)
Wolfowitz Staphylococcus	1	0	0	0	0	0	0	0	1 (1.9)
Staphylococcus aureus	1	1	0	0	0	0	1	0	3 (5.6)
Staphylococcus epidermidis	1	1	0	0	0	0	0	0	2 (3.7)
Staphylococcus heads	0	0	0	0	0	0	0	1	1 (1.9)
Acinetobacter baumannii	1	0	0	5	1	0	0	0	7 (13.0)
Pang Proteus	1	0	0	0	0	0	0	0	1 (1.9)
Bacillus cereus	1	0	0	0	0	0	0	0	1 (1.9)
Ruben A. baumannii	1	0	0	0	0	0	0	0	1 (1.9)
Proteus mirabilis	1	0	0	0	0	0	1	0	2 (3.7)
Enterobacter cloacae	0	1	0	1	0	0	0	0	2 (3.7)
Streptococcus pneumoniae	0	0	0	1	0	0	0	0	1 (1.9)
Viridans	0	0	0	1	0	0	0	0	1 (1.9)
Streptococcus mitis	1	0	0	0	0	0	0	0	1 (1.9)
Streptococcus agalactiae	0	0	0	0	0	0	1	0	1 (1.9)
Klebsiella pneumoniae	0	0	0	6	0	0	0	0	6 (11.1)
Enterococcus faecalis	0	0	0	0	1	0	0	0	1 (1.9)
Candida albicans	0	0	0	4	0	0	0	0	4 (7.4)
Candida tropicalis	0	0	0	0	2	0	0	0	2 (3.7)
Portugal candida	0	0	0	0	1	0	0	0	1 (1.9)
Morganella morganii	0	0	0	0	0	0	1	0	1 (1.9)
Non-de-lux coli	0	0	0	0	0	0	1	0	1 (1.9)
Aspergillus	0	0	0	1	0	0	0	0	1 (1.9)
Total	14 (25.9)	4 (7.4)	1 (1.9)	19 (35.2)	7 (13.0)	1 (1.9)	6 (11.1)	2 (3.7)	54 (100.0)

### Management

A total of 81 patients received blood transfusion therapy. The most common blood transfusion therapy involved red blood cells; this was followed by plasma transfusion. Seventy-five patients received a median transfusion of 3.5 units (2.0–6.0) of red blood cells. Forty patients received a median transfusion of 4.0 units (3.0–8.0) of plasma. Nine patients received transfusions of whole blood or platelets. Thirty-six patients received ventilator-assisted breathing therapy. Regarding the treatment of crush injuries, fasciotomies were performed in 25 cases, six of which were performed in affected hospitals and 19 of which were performed in backup hospitals. Amputations were performed in 12 cases, four of which occurred in affected hospitals and eight of which occurred in backup hospitals. No patient received renal replacement therapy.

#### Rehabilitation

A total of 1418 (70.5%) patients had limb dysfunction, and rehabilitation records were found for 852 (60.1%) of this group (Table 5). The median interval before receiving rehabilitation was 1 week (IQR 0 week), and the median duration of rehabilitation was 3 weeks (IQR 6 weeks). A total of 599 (70.3%) patients received rehabilitation in medical institutions, and 148 (17.4%) patients recovered in non-medical institutions. A total of 806 (97.9%) patients received conventional rehabilitation, 135 (16.4%) received neuropsychological rehabilitation, 61 (7.4%) received technological interventions, and two (0.2%) patients received language and swallowing therapy. A total of 648 (78.7%) patients received one rehabilitation program, 167 (20.3%) patients received two rehabilitation programs, and 8 (1.0%) received three programs. A total of 508 hospitalized patients needed rehabilitation- assistant devices, 145 (28.5%) used wheelchairs, 77 (15.2%) used walkers, 273 (53.7%) used crutches, and 13 (2.6%) used canes.

**Table 5:** Analysis of hospitalized patients with limb dysfunction.

Types of limb dysfunction	Number (%)
Pain	1304 (92.0)
Pedestrian barriers	660 (46.5)
Up and down stairs barriers	622 (43.9)
Joint movement barriers	573 (40.4)
Bathing barriers	558 (39.4)
Toilet barriers	519 (36.6)
Personal hygiene limited	418 (29.5)
Bed chair transfer barriers	347 (24.5)
Balance barriers	289 (20.4)
Dressing barriers	288 (20.3)
Muscle weakness	177 (12.5)
Sensory dysfunction	91 (6.4)
Eating barriers	56 (3.9)
Dystonia	20 (1.4)
Bladder control disorders	19 (1.3)
Anal control disorders	11 (0.8)

### Outcomes

Of the 2010 patients, 93 were critically ill, and 240 were seriously ill. A total of seven patients died during hospitalization, and their mean age was 45.3±14.4 years (median 43, range 25–72 years). The age distribution of deceased individuals is presented in Table 2. All seven of these deceased patients died of severe traumatic brain injuries; five of this group died within 24 h after the earthquake, one died 72 h after the earthquake, and one died 2 months after the earthquake.

## Discussion

This is the first epidemiological study, which was organized by the Health Administrative Department, adopted a systematic approach to understanding the earthquake-related injuries and treatment of all hospitalized seismic patients. These data can make an important contribution to the efforts of governments and aid organizations to treat and organize the treatment of future earthquake-related injuries. These data on the Lushan earthquake differ from data on previous earthquakes in several respects. First, the treatment of all critically injured patients was centralized, and the rescue of earthquake survivors was effective. Second, we found more jumping injuries and fewer crush injuries. Third, hospital infection control and rehabilitation specialists participated during the early phase of the response.

Three earthquakes with magnitudes over 7.0 on the Richter scale have occurred in China in the past 5 years. Due the massive number of injured individuals and the inadequacy of the treatment available in local areas, most people injured in the Wenchuan and Yushu earthquakes were transported to other areas [8], [9]. Although the dispersion of patients decreased the burden on local treatment facilities, it also made it difficult to conduct epidemiological research because injured people were transported to too many hospitals to allow investigation. However, the Lushan earthquake, which also scored higher than 7.0 on the Richter scale, affected an area with a relatively low population density, and all injured people were treated at the 140 hospitals within the local province under the aegis of the Health Administrative Department of Sichuan province. This situation allowed us to conduct this epidemiological study.

The rescue operation following the earthquake rested on the four centralization principles [2]. The Sichuan Provincial Health Administration organized the rescue and, during the second day after the earthquake, the National Health and Family Planning Commission selected the most highly qualified medical specialists with seismic experience to form a national expert medical team to guide the rescue operations. From April 21 to May 13, this team of medical experts screened every critically ill patient, and all critically injured patients were transferred to three larger general hospitals in Chengdu city (the West China Hospital, Sichuan Provincial People’s Hospital, and Chengdu Military Region General Hospital) within a week after the earthquake. As these three hospitals had previously treated many injured patients in the aftermath of the Wenchuan and Yushu earthquakes, they had considerable relevant experience. Due to transportation problems, the last two severely injured patients, who had suffered brain injuries, were transferred by aircraft, accompanied by a treatment team of experts, from Ganzi People’s Hospital to West China Hospital on April 28.

Similar to the previous earthquakes [10]–[14], the most common types of injury suffered in the Lushan disaster were bone fractures, brain injuries, and chest injuries, and the most common cause of death was brain injury. Seven hospitalized patients who had suffered severe brain injuries died. Unlike the Wenchuan earthquake [15], most of the areas affected by the Lushan earthquake were remote rural and no tall buildings collapsed. Injured patients were rescued immediately. This minimized the number of crush injuries; only 1.7% of the injured patients suffered crushed limbs, and none was treated with renal replacement treatment.

Post-earthquake infections are common and associated with a high mortality rate [16]–[18]. About 500 injured patients were admitted to Lushan County Hospital, which accepted the greatest number of injured patients. All severely injured patients were admitted to the back up hospitals, and the most common reasons for admission to Lushan County Hospital were mild injuries and common diseases. Nearly 1000 outpatients sought treatment at Lushan County Hospital, which was twice the usual number. As the integrity of the hospital building was difficult to evaluate, all patients were treated in a temporary tent characterized by poor conditions and the absence of essential surgical equipment and infection-control measures. In contrast, the West China Hospital of Sichuan University participated in three emergency medical rescues related to earthquakes (Wenchuan, Yushu, and Lushan) in the past 5 years, which added to their experience in this domain [19]–[21]. After the Lushan earthquake, supplementary provisions were added to the original West China Hospital Earthquake casualty triage disposal process. To protect against infection, the hospital classified patients according to injury, site, and infection risk. Experts in the hospital made daily rounds to visit critically ill patients in the wards and adjust their medication. Hand hygiene facilities and the placement of obvious warnings near the bed units and medical workspaces in the ICU were improved.

As the magnitude of the Lushan earthquake was significantly lower than that of the Wenchuan earthquake, the numbers of patients requiring amputation and suffering from paraplegia as a result of their wounds were relatively small, 12 and 20, respectively. However, many patients who suffered fractures and peripheral nerve injuries required rehabilitation [22], [23]. As the rehabilitation resources in Sichuan province have developed rapidly since the Wenchuan earthquake in 2008, this area was prepared for the needs of these patients [24], [25]. Indeed, most of the injured patients with limb dysfunction received rehabilitation within 1 week after the earthquake. A total of 70.3% of the rehabilitation therapy was performed at health facilities. Follow-up research will determine the long-term effects of early rehabilitation interventions.

The treatment of injured children after disasters requires improvement [26], [27]. The expert team reported that the follow-up treatment provided by some hospitals for the traumatic injuries suffered by children was not appropriate. One major reason for this situation was the relative scarcity of pediatric orthopedists and pediatric surgeons. Indeed, care for injured pediatric patients provided by specialists in orthopedics and traumatology was based on experience with adults. Similar to the situation following the Wenchuan earthquake [28], a large proportion of injured patients were elderly individuals (25.2% of all injured patients were older than 60 years). Patients older than 80 years suffered more hip and thigh fractures, pelvis fractures, and chest injuries. The elderly were also more likely to suffer pulmonary infection. As elderly individuals are more likely to suffer from underlying diseases, to be less able to compensate for injuries to different organs, and to be more vulnerable to new complications [29], [30], more attention should be devoted to enhancing geriatric treatment in the future.

The injuries of some patients were indirectly caused by the earthquake. Due to the severe trauma caused by the Wenchuan earthquake, the local residents reacted with great fear to the Lushan earthquake. Many residents who were not in the epicenter of the earthquake jumped from tall buildings, and 371 patients were injured as a result; this group included 17 (13.4%) and 61 (22.5%) of those who were critically and severely injured, respectively. Sichuan Agricultural University is the only university in Ya’an. Although the earthquake fractured the building of the university, it did not collapse, and more than 100 students were slightly injured while escaping. Seven were injured by jumping from the building and were sent to Ya’an People’s Hospital [31]. Thus, education about how to escape from a building during an earthquake should be enhanced to minimize the indirect damage caused by disasters.

The situation of each earthquake is unique. Yet, knowledge about the injuries caused by each disaster and how to treat them can improve the organization, treatment, and rehabilitation available for future disasters. Moreover, improving disaster-related education should also reduce secondary injuries.

## References

[1] Zhang L, Liu X, Li Y, Liu Y, Liu Z, et al (2012) Emergency medical rescue efforts after a major earthquake: lessons from the 2008 Wenchuan earthquake. Lancet 379: 853–61. 22386038 10.1016/S0140-6736(11)61876-X

[2] Shen J, Kang J, Shi Y, Li Y, Li Y, et al (2012) Lessons learned from the Wenchuan earthquake. J Evid Based Med 5: 75–88. 23557471 10.1111/j.1756-5391.2012.01176.x

[3] Kang P, Zhang L, Liang W, Zhu Z, Liu Y, et al (2012) Medical evacuation management and clinical characteristics of 3,255 inpatients after the 2010 Yushu earthquake in China. J Trauma Acute Care Surg 72: 1626–33. 22695432 10.1097/TA.0b013e3182479e07

[4] Tang B, Zhang L (2013) Ya’an earthquake. Lancet 381: 1984–5. 10.1016/S0140-6736(13)61201-5 23746896

[5] Ouyang Y (2013) Earthquake tests China’s emergency system. Lancet 381: 1801–2. 23717832 10.1016/S0140-6736(13)61105-8 PMC7138371

[6] Zhang L, Chen XM (2014) Enhancing the scientific management of earthquake disaster rescue: Further improvements to the state level of disaster medical care. Chinese Medical Journal 127: 995–7. 24571908

[7] Lushan earthquake medical expert team (2013) The Earthquake Wounded Condition Assessment and Management Standards. Chinese Medical Journal 93: 1527–8.

[8] Chen J, Zhao WH, Xian M, Lu J, Liang Z (2009) Trans-province transfer of 10373 patients injured in Wenchuan earthquake. Chin J Evid Based Med 9: 1267–71. 10.1111/j.1756-5391.2009.01053.x 21349026

[9] Liu X, Liu Y, Zhang L, Liang W, Zhu Z, et al (2013) Mass aeromedical evacuation of patients in an emergency: experience following the 2010 yushu earthquake. J Emerg Med 45: 865–71. 23993933 10.1016/j.jemermed.2013.05.054

[10] de Bruycker M, Greco D, Annino I, Stazi MA, de Ruggiero N, et al (1983) The 1980 earthquake in southern Italy: rescue of trapped victims and mortality. Bull World Health Organ 61: 1021–5. 6609007 PMC2536241

[11] Oda J, Tanaka H, Yoshioka T, Iwai A, Yamamura H, et al (1997) Analysis of 372 patients with Crush syndrome caused by the Hanshin-Awaji earthquake. J Trauma 42: 470–5. 9095115 10.1097/00005373-199703000-00015

[12] Sever MS, Erek E, Vanholder R, Akoğlu E, Yavuz M, et al (2001) The Marmara earthquake: epidemiological analysis of the victims with nephrological problems. Kidney Int 60: 1114–23. 11532107 10.1046/j.1523-1755.2001.0600031114.x

[13] van der Tol A, Hussain A, Sever MS, Claus S, Van Biesen W, et al (2009) Impact of local circumstances on outcome of renal casualties in major disasters. Nephrol Dial Transplant 24: 907–12. 18842675 10.1093/ndt/gfn557

[14] Ardagh MW, Richardson SK, Robinson V, Than M, Gee P, et al (2012) The initial health-system response to the earthquake in Christchurch, New Zealand, in February, 2011. Lancet 379: 2109–15. 22510397 10.1016/S0140-6736(12)60313-4

[15] Jiang J, Li Y, Huang X, Li B, Su L, et al (2012) Lessons learnt from the Wenchuan earthquake: performance evaluation of treatment of critical injuries in hardest-hit areas. J Evid Based Med 5: 114–23. 23672218 10.1111/j.1756-5391.2012.01186.x

[16] Miskin IN, Nir-Paz R, Block C, Merin O, Burshtein S, et al (2010) Antimicrobial therapy for wound infections after catastrophic earthquakes. N Engl J Med 363: 2571–3. 21175337 10.1056/NEJMc1005578

[17] Wang Y, Hao P, Lu B, Yu H, Huang W, et al (2010) Causes of infection after earthquake, China, 2008. Emerg Infect Dis 16: 974–5. 20507749 10.3201/eid1606.091523 PMC3086233

[18] Ran YC, Ao XX, Liu L, Fu YL, Tuo H, et al (2010) Microbiological study of pathogenic bacteria isolated from paediatric wound infections following the 2008 Wenchuan earthquake. Scand J Infect Dis 42: 347–50. 20095936 10.3109/00365540903510682

[19] Chen E, Deng L, Liu Z, Zhu X, Chen X, et al (2011) Management of gas gangrene in Wenchuan earthquake victims. J Huazhong Univ Sci Technolog Med Sci 31: 83–7. 21336729 10.1007/s11596-011-0155-3

[20] Zhang H, Zeng JW, Wang GL, Tu CQ, Huang FG, et al (2013) Infectious complications in patients with crush syndrome following the Wenchuan earthquake. Chin J Traumatol 16: 10–5. 23384864

[21] Tao C, Kang M, Chen Z, Xie Y, Fan H, et al (2009) Microbiologic study of the pathogens isolated from wound culture among Wenchuan earthquake survivors. Diagn Microbiol Infect Dis 63: 268–70. 19135331 10.1016/j.diagmicrobio.2008.11.009

[22] Raissi GR (2007) Earthquakes and rehabilitation needs: experiences from Bam, Iran. J Spinal Cord Med 30: 369–72. 17853660 10.1080/10790268.2007.11753954 PMC2031928

[23] Landry MD, Quigley A, Nakhle A, Nixon SA (2010) Implications of a gap between demand and supply for rehabilitation in post-earthquake Haiti. Physiother Res Int 15: 123–5. 20812312 10.1002/pri.488

[24] Li Y, Pan F, Li Y (2009) Analysis of rehabilitation needs, measures taken, and their effectiveness for the wounded following the Wenchuan Earthquake. J Evid Based Med 2: 258–64. 21349024 10.1111/j.1756-5391.2009.01045.x

[25] Li S, He C (2013) Early rehabilitation prevents disability after earthquake: A letter to international rehabilitation colleagues. J Rehabil Med 45: 603. 23681118 10.2340/16501977-1176

[26] Mace SE, Bern AI (2007) Needs assessment: are disaster medical assistance teams up for the challenge of a pediatric disaster? Am J Emerg Med 25: 762. 17870478 10.1016/j.ajem.2006.12.011

[27] Sugihara G, Suda S (2011) Need for close watch on children’s health after Fukushima disaster. Lancet 378: 485–6. 10.1016/S0140-6736(11)61250-6 21821184

[28] Zhang L, Fu P, Wang L, Cai G, Zhang L, et al (2012) The clinical features and outcome of crush patients with acute kidney injury after the Wenchuan earthquake: differences between elderly and younger adults. Injury43: 1470–5. 10.1016/j.injury.2010.11.036 21144512

[29] Emily YY (2008) The untold stories of the Sichuan earthquake. Lancet 372: 359. 18675675 10.1016/S0140-6736(08)61141-1

[30] Bartels SA, VanRooyen MJ (2012) Medical complications associated with earthquakes. Lancet 379: 748–57. 22056246 10.1016/S0140-6736(11)60887-8

[31] China Earthquake Administration website. Available: http://www.cea.gov.cn/publish/dizhenj/468/553/100342/100343/20130524175506536710194/index.html. Accessed 2014 Apr 25.

